# Miss Agnes Jones' Great Work

**Published:** 1918-03-09

**Authors:** Arthur Stanley


### LIVERPOOL A PIONEER IN NURSING.

### Miss Agnes Jones' Great Work.

BY THE HON. SIR ARTHUR STANLEY, G.B.E., C.B.*

There are three dates in the history of nursing that are really of paramount importance. The first date is when Miss Florence Nightingale set out for the Crimea. The second date is when, after the Crimea, a fund was raised to commemorate the efforts of Miss Florence Nightingale and her devoted band of workers in the Crimea. That fund was called the "Nightingale Fund." It was given to Miss Florence Nightingale to do whatever she liked with, and she decided with it to found the Nightingale School of Nurses at St. Thomas's Hospital, London. That was founded in 1860, and I am proud that I am now connected with St. Thomas's Hospital, in which there is the Nightingale School of Nurses.

But a date which Miss Florence Nightingale herself, I think, regarded almost as important as either of those other two is May 16, 1865, when Miss Agnes Jones came down to Liverpool, the first one to establish nursing in a workhouse infirmary. I recommend anyone who has never read about those times to do so. Anything more appalling than the circumstances which Miss Agnes Jones had to deal with when she came to Liverpool it is almost impossible to imagine. There is one thing to remember in connection with this matter—namely, that a name as honoured as that of Miss Agnes Jones or Miss Nightingale is that of Mr. Rathbone, of Liverpool. It was entirely due to his generosity that Miss Agnes Jones was able to come to Liverpool. There was no money found by the State for the experiment, which, I believe, cost something like £1,200 a year. It was entirely due to Mr. Rathbone's initiative and to his generosity that the whole system of general workhouse nursing was set up.

One fact struck me when I was looking the other day at some of Miss Agnes Jones's letters. It was quite a common thing for there to be two or three or four patients in one workhouse bed; the numbers in the infirmary were always greater than the number of beds. It was a usual thing for a man to wear his shirt for seven weeks without being washed, and the bed-clothes were very seldom washed under months. Just think what that one lady had to contend with—all the ignorance, all the prejudice, and all the absolute physical dirt. If all this is considered it will be realised that the date when Agnes Jones came to Liverpool and started nursing in a workhouse infirmary is one that ought to be noted in letters of gold in the history of nursing. She only came to Liverpool in 1865, and she died at the beginning of 1868, but in that time she did an imperishable work, and a work with which the name of Liverpool will be associated for ever. That is the reason why I want Liverpool to be the first centre of the College of Nursing. Liverpool has always been a pioneer and a place for experiments which are not dared to be attempted in London, simply because London knows that Liverpool is very much in advance in intelligence and will understand them.

* At the Liverpool meeting of the College of Nursing, February 22, 1918.

